# AlliumDB: a central portal for comparative and functional genomics in *Allium*

**DOI:** 10.1093/hr/uhad285

**Published:** 2023-12-29

**Authors:** Pengtao Yang, Yu Yuan, Chao Yan, Yue Jia, Qi You, Lingling Da, Ao Lou, Bingsheng Lv, Zhonghua Zhang, Yue Liu

**Affiliations:** Engineering Laboratory of Genetic Improvement of Horticultural Crops of Shandong Province, College of Horticulture, Qingdao Agricultural University, Qingdao 266109, China; Engineering Laboratory of Genetic Improvement of Horticultural Crops of Shandong Province, College of Horticulture, Qingdao Agricultural University, Qingdao 266109, China; Engineering Laboratory of Genetic Improvement of Horticultural Crops of Shandong Province, College of Horticulture, Qingdao Agricultural University, Qingdao 266109, China; Engineering Laboratory of Genetic Improvement of Horticultural Crops of Shandong Province, College of Horticulture, Qingdao Agricultural University, Qingdao 266109, China; Key Laboratory of Plant Functional Genomics of the Ministry of Education/Jiangsu Key Laboratory of Crop Genomics and Molecular Breeding/Co-Innovation Center for Modern Production Technology of Grain Crops, College of Agriculture, Yangzhou University, Yangzhou 225009, China; College of Life Science, Northwest Normal University, Lanzhou 730070, China; Engineering Laboratory of Genetic Improvement of Horticultural Crops of Shandong Province, College of Horticulture, Qingdao Agricultural University, Qingdao 266109, China; Engineering Laboratory of Genetic Improvement of Horticultural Crops of Shandong Province, College of Horticulture, Qingdao Agricultural University, Qingdao 266109, China; Engineering Laboratory of Genetic Improvement of Horticultural Crops of Shandong Province, College of Horticulture, Qingdao Agricultural University, Qingdao 266109, China; Engineering Laboratory of Genetic Improvement of Horticultural Crops of Shandong Province, College of Horticulture, Qingdao Agricultural University, Qingdao 266109, China

## Abstract

The genus *Allium* belongs to the botanical family Amaryllidaceae and includes economically important crops such as onion, garlic, bunching onion, and leek, used as vegetables, spices, and traditional medicines. The large sizes of *Allium* genomes hamper the genetic dissection of agronomically important traits and molecular breeding. With the growing accumulation of genomic, resequencing, transcriptome, and phenotypic data, the demand for an integrative *Allium* database is increasing. Here we present a user-friendly database, AlliumDB (https://allium.qau.edu.cn), as a functional genomics hub integrating public and in-house data. The database contains all currently available nuclear and organelle genomes for *Allium* species, with genes comprehensively annotated based on Gene Ontology (GO) and Kyoto Encyclopedia of Genes and Genomes (KEGG) analyses, orthology, gene families, protein families (Pfam), and non-coding RNA families (Rfam). Transcriptome and variation profiles are integrated into dynamic visualization tools. We took phenotypic photographs and generated trait records for hundreds of *Allium* germplasms collected worldwide, which are included in the database. We incorporated JBrowse for the visualization of gene structures, RNA sequencing data, and variation data. Analysis tools such as the basic local alignment search tool (BLAST), sequence fetch, enrichment, and motif analyses are available to explore potential gene functions. This database incorporates comprehensive *Allium* genotypic and phenotypic datasets. As the community assembles new genomes and generates resequencing data for *Allium* germplasms, the database will be improved and continuously updated with these multi-omics data and comparative genomic studies. We expect the AlliumDB database to become a key resource for the study of *Allium* crops.

## Introduction

*Allium* L. is a large genus of monocotyledonous plants, belonging to the family Amaryllidaceae in the order Asparagales. The genus comprises more than 900 species distributed throughout the world in temperate, tropical, and semi-arid regions, mainly in Asia, North America, Europe, and northern Africa [1, 2]. *Allium* plants usually produce bulbs, which are of economic importance because of their edible fleshy scale leaves, unique flavor, and nutritional value. Among *Allium* plants, several crop species, including onion (*A. cepa*), garlic (*A. sativum*), bunching onion (*A. fistulosum*), leek (*A. ampeloprasum*), Chinese chive (*A. tuberosum*), and chive (*A. schoenoprasum*), are widely cultivated. *Allium* species are also often used in traditional medicine due to their anticarcinogenic, antibiotic, antithrombotic, and cardioprotective properties [3]. More than 141 million tons of *Allium* crops were produced worldwide in 2021 (www.fao.org, accessed in 2023).

Despite the economic importance of *Allium* crops, their genetic study and breeding are hampered by their biennial life cycle, cross-pollination requirement, and inbreeding depression [4], as well as their relatively large genomes (10–30 Gb). Large genomes are difficult to sequence and assemble, which has slowed genomic research in *Allium* species. With the development of single-molecule real-time (SMRT) sequencing and nanopore long-read sequencing technologies, however, scientists are beginning to make breakthroughs in the assembly and study of large genomes. In recent years, the chromosome-level *Allium* genome assemblies for *A. sativum*, *A. cepa*, and *A. fistulosum* have been completed [5–8]. Many transcriptome and resequencing datasets have also been generated from various *Allium* species to explore the molecular mechanisms underlying key traits, such as bulb development [9], flavonoid biosynthesis [10], male sterility [11], and stress tolerance [12].

**Figure 1:**
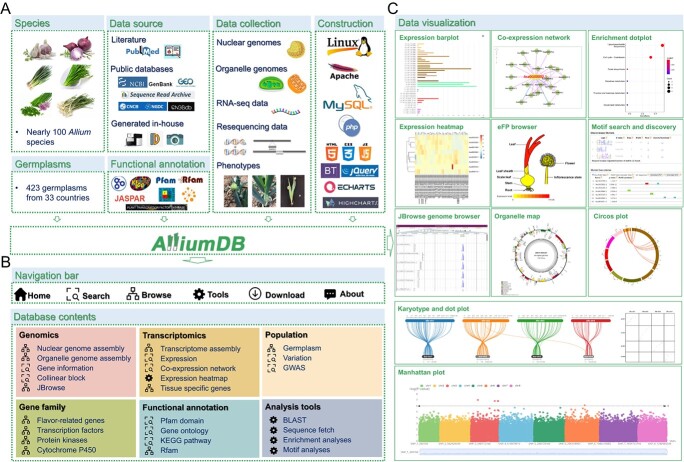
Overview of AlliumDB. **A** Data sources, contents, and database construction. AlliumDB covers a wide range of *Allium* species and germplasms and incorporates rich publicly available and in-house data types, including genomes, gene functional annotations, transcriptomes, variations, and manually recorded phenotypes. AlliumDB was constructed based on a standard LAMP system. **B** Database architecture. The five main functions of the navigation bar (Search, Browse, Tools, Download, and About) are related to the database contents. **C** Diversified data visualization. AlliumDB provides a variety of visualization tools for rich data, including bar plots, heat maps, an electronic fluorescent pictographic (eFP) browser for expression patterns, networks displaying co-expression relationships, dot plots for enrichment analyses, motif logos for motif analyses, organelle genome maps, karyotypes, dot and Circos plots for collinearity, Manhattan plots for genome-wide association studies (GWAS) and JBrowse for RNA-seq and resequencing data.

A comprehensive scientific database can be generated by integrating genome sequences and functional annotations, multi-omics data, germplasm resource information, and convenient analysis tools, providing researchers with consistent information, accelerating scientific breakthroughs, and promoting breeding innovations. Increasing numbers of genus- and family-level databases have been established and include multiple horticultural species for comparative and functional genomics research, such as the Cucurbit Genomics Database (CuGenDB) [13], the Sol Genomics Network (SGN) [14], the Citrus Pan-Genome to Breeding Database (CPBD) [15], Portal of Juglandaceae (PJU) [16], The Vegetable Information Resource (TVIR) [17], traditional Chinese Medicine Plant Genomes (TCMPG) [18], and the Heat Shock Factor Database (HsfDB) [19]. These databases have accelerated functional and comparative genomics research and molecular breeding in their corresponding fields.

*Allium* species are an understudied group of plants that are now receiving widespread attention from the scientific community [20, 21]. Copious data are rapidly being produced for these species from various omics experiments. For large genomes, an integrated platform combining comparative genomics and multi-omics data is particularly important for the elucidation of complex molecular mechanisms. Here we developed a user-friendly database, named AlliumDB, which integrates large-scale *Allium* data from public and in-house databases and enables users to store, analyze, visualize, and mine these complex biological datasets. AlliumDB includes multi-omics data, such as genome, resequencing, transcriptome, and proteome data, and integrates rich germplasm resources with abundant manually collected phenotypic data. AlliumDB also provides many easy-to-use analytical tools and a download center that enables effective utilization of the data and provides a convenient means to retrieve and analyze functional genomic information. AlliumDB will facilitate future scientific research and the molecular breeding of *Allium* crops.

**Table 1:** Summary of AlliumDB constituent datasets.

**Category**	**Details**	**Description**
Assembly	Nuclear genome assembly	Three species
	Organelle genome assembly	89 species, five mitochondrion genomes, 221 chloroplast genomes
	Transcriptome assembly	Four species
Gene family	Flavor-related genes	90 genes in *A. sativum*
		109 genes in *A. cepa*
		115 genes in *A. fistulosum*
	Transcription factors/transcriptional regulators	2600 genes in *A. sativum*
		2698 genes in *A. cepa*
		2801 genes in *A. fistulosum*
	Protein kinases	1537 genes in *A. sativum*
		1253 genes in *A. cepa*
		1318 genes in *A. fistulosum*
	CYP450s	604 genes in *A. sativum*,
		437 genes in *A. cepa*
		477 genes in *A. fistulosum*
Functional annotation	GO	28 317 genes in *A. sativum*
		23 748 genes in *A. cepa*
		29 984 genes in *A. fistulosum*
	KEGG	39 000 genes in *A. sativum*,
		24 261 genes in *A. cepa*
		14 203 genes in *A. fistulosum*
	Pfam domains	37 090 genes in *A. sativum*
		41 882 genes in *A. cepa*
		35 013 genes in *A. fistulosum*
	Nr	49 428 annotations in *A. sativum*
		73 318 annotations in *A. cepa*
		44 770 annotations in *A. fistulosum*
	Swiss-Prot	38 809 annotations in *A. sativum*
		37 730 annotations in *A. cepa*
		33 130 annotations in *A. fistulosum*
	trEMBL	49 483 annotations in *A. sativum*
		72 679 annotations in *A. cepa*
		44 678 annotations in *A. fistulosum*
	Ortholog in *Arabidopsis*	47 736 genes in *A. sativum*
		46 762 genes in *A. cepa*
		40 072 genes in *A. fistulosum*
	Ortholog group	21 254 groups
	ncRNA	21 920 annotations in *A. sativum*
		17 740 annotations in *A. cepa*
		12 034 annotations in *A. fistulosum*
	Co-expression	405 196 positive and 141 360 negative gene pairs in *A. sativum*
		6 977 016 positive and 193 968 negative gene pairs in *A. cepa*
		673 005 positive and 197 756 negative gene pairs in *A. fistulosum*
Comparative genomics	Collinear blocks	111 blocks in *A. sativum* vs *A. cepa*
		647 blocks in *A. sativum* vs *A. fistulosum*
		260 blocks in *A. cepa* vs *A. fistulosum*
Omics data	Transcriptome	746 samples from seven species
	Proteome	Sourced from seven publications
	Variations	336 accessions
Germplasm	Germplasms	423 *Allium* germplasms from 33 countries, 17 species
	Photographs	1317 photographs
	Traits	Nine traits (tillering, leaf wax, bolting, leaf shape, leaf gesture, orientation of leaf, node density, length of pseudostem, germination rate)

## Results

### Database overview

AlliumDB is a comprehensive functional genomics platform for the *Allium* genus, integrating genome, gene functional annotation, genome variation, gene expression, protein abundance, phenotypic, and comparative genomic data (Fig. 1A and Table 1). At present, AlliumDB contains three nuclear genomes from three *Allium* species and 227 organelle genomes from 89 *Allium* species, 746 transcriptome deep sequencing (RNA-seq) datasets (including 49 datasets newly generated by our laboratory from different tissues of onion and Welsh onion) from seven *Allium* species, 420 genotype-by-sequencing (GBS) or resequencing datasets, proteomic data collected from seven articles, 1317 photographs detailing phenotypes, and phenotypic information for nine traits recorded manually from 423 germplasms in 33 countries. For each nuclear genome, AlliumDB provides multiple functional annotation tools. In total, 167 516, 109 669, and 166 840 genes are annotated by the Non-redundant (Nr), Swiss-Prot, and trEMBL databases, respectively. In addition, 82 049 genes are annotated with Gene Ontology (GO) terms; 77 464 genes are annotated with Kyoto Encyclopedia of Genes and Genomes (KEGG) pathway terms; the proteins encoded by 113 985 genes are annotated with Pfam domains; and 51 694 loci are annotated as non-coding RNA (ncRNA). We determined best hits with *Arabidopsis* (*Arabidopsis thaliana*) orthologs for 134 570 genes; we provide 21 254 orthologous groups in three *Allium* genomes and two model species [*Arabidopsis* and rice (*Oryza sativa*)]. We also assigned co-expression relationships for 8 588 301 gene pairs. Multiple gene families are stored in AlliumDB, including 314 flavor-related genes, 8099 genes encoding transcription factors or transcriptional regulators, 4108 genes encoding protein kinases, and 1518 cytochrome P450 (CYP450) genes. For comparative genome analyses, we identified 1018 collinear blocks among three *Allium* species.

### Database functions

AlliumDB provides a convenient and centralized means of retrieving, analyzing, and visualizing gene functions and multi-omics data from *Allium* species. The Search, Browse, Tools, and Download functions were designed to be user-friendly, with hyperlinks and dynamic charts (Fig. 1B and C).

#### Search

The Search function contains gene information, functional annotations, co-expression networks, multi-omics data, and collinear blocks. Detailed gene information can be obtained by using a specific locus identifier (locus ID) as search input. Chromosomal locations and keywords are also permitted inputs for retrieving relevant genes, and users can access the relevant gene information page by clicking on a gene ID. The information page for each gene shows its location, gene model (in an embedded JBrowse), transcript and protein sequences, best hits in the Nr, Swiss-Prot, trEMBL, and Arabidopsis Information Resource (TAIR) databases, orthologous groups, Pfam domains of the encoded protein, GO and KEGG annotations, and expression patterns in different tissues (as bar plots and eFP browser). In addition, GO, KEGG, and Pfam searches can be performed with an ID or keyword to retrieve a list of genes with the corresponding annotation. The genes showing a positive or negative co-expression with a gene of interest can be extracted using the locus ID. The results pages show the network view and relationship table, with annotations and Pearson’s correlation coefficients. The bottom of the results page contains links to analysis tools, such as expression heat map displays and GO, KEGG, and gene set enrichment analyses; when a user clicks on a tool link, the co-expressed genes are automatically loaded into the tool as an input. The expression values [expressed as fragments per kilobase of transcript per million mapped reads (FPKM)] and sequence variations for a specific gene derived from multiple collected omics datasets are included when available, and the corresponding sample/accession information is organized and displayed in the search result page. The GWAS (genome-wide association study) page reports those single nucleotide polymorphisms (SNPs) that show a statistically significant association with a trait of interest in the form of Manhattan plots and annotation tables. Collinear blocks in the three *Allium* nuclear genome assemblies can be searched by chromosome or gene ID, with each gene pair and corresponding E-value shown by clicking on the alignment ID. The synteny visualization tool SynVisio is provided for different types of synteny visualizations, including hive plots for synteny between chromosomes, dot plots for collinearity between two genomes, and scatterplots for identified signal strength.

#### Browse

The Browse function provides access to nuclear and organelle genome assemblies; transcriptome assemblies; gene families, including flavor-related genes, transcription factor, and transcriptional regulator genes, protein kinase genes, and CYP450 genes; ncRNA families; tissue-specific genes; proteome data; and a JBrowse genome browser and germplasm information. The nuclear genome browse page offers an introduction to the species and their published genome assemblies. For each genome assembly, a link to search the gene information associated with this genome is provided. The organelle genomes can be browsed by organism, genome name, size, GenBank accession number, author, sequence, and graphical map. For each transcriptome assembly, a link to search the annotation and expression of a given unigene is provided. On the gene family (transcription factor and transcriptional regulator genes, protein kinase genes, and CYP450 genes) and Rfam page, users can select a species name to access a list of the subfamilies and gene members in that species. On the tissue-specific genes page, a cutoff of Tau index [22] can be set for a customized search. All gene IDs in the database are hyperlinked to allow users to jump to the gene information page. The proteome page shows the relevant published articles and protein information. All available genomes and gene models were imported into JBrowse, which also contains tracks for multi-omics data, including expression levels and variants with a single-base resolution.

**Figure 2:**
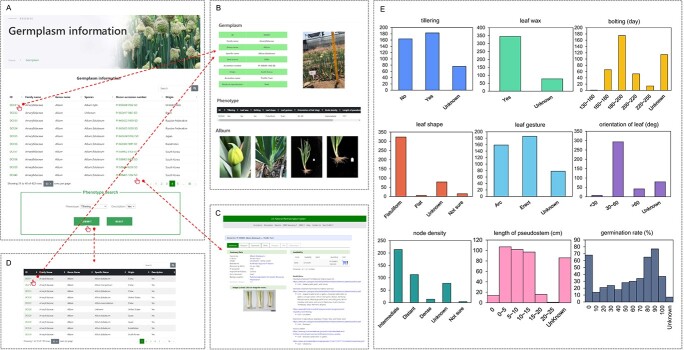
The germplasm information module in AlliumDB. **A** Screenshot of the germplasm page. **B** Screenshot of a germplasm detail information page. **C** Screenshot showing the source of the accession DC040. **D** Screenshot of germplasm results after a phenotypic search. **E** Summary of phenotypic records in AlliumDB. The *y*-axes represent the number of germplasms.

We collected *Allium* germplasms worldwide for phenotypic surveys and are planning to continuously upload phenotypic records and photographs to AlliumDB as they become available (Fig. 2). The germplasm browse page (Fig. 2A) shows the origin of each accession and seed source with a hyperlink to relevant web pages (Fig. 2C). A more detailed information page (Fig. 2B), including photographs and manually collected traits (Fig. 2E), can be accessed by clicking on the germplasm ID. A search form is provided for querying germplasm by phenotype (Fig. 2A and D). We are generating resequencing data for these germplasms and are updating the associated genotypes and phenotypes in AlliumDB.

#### Tools

The Tools function contains several convenient and useful online tools for displaying and analyzing data, including BLAST, enrichment analysis, motif analysis, expression heat maps, and sequence fetch. The BLAST interface stores the coding sequence and protein sequence of the assembled *Allium* species and *Arabidopsis*. An enrichment analysis interface provides enrichment of GO terms, KEGG pathways, and gene families with different parameters for selection. The expression heat map tool can receive a batch list of gene IDs as an input to draw the corresponding heat map plots. The sequence fetch tool can be used to obtain FASTA format sequences for coding sequences, proteins, and genes; the 1-, 2-, or 3-kb sequences upstream of query genes; and the sequence from a certain position on a chromosome. Quick links are provided to jump directly to a BLAST/motif search tool page. The motif analysis allows users to scan for and discover motifs in sequences of interest, either by pasting a sequence into the text box or by uploading a FASTA file. Each analysis in the Tools function will provide a job ID to enable users to repeatedly view the results using the result review function, and the output files are downloadable from the analysis result pages.

#### Download

The resources in AlliumDB can be downloaded for personal use. Quick download links are provided.

### Case study: functional and conservation analysis of *CYP75B* using AlliumDB

**Figure 3:**
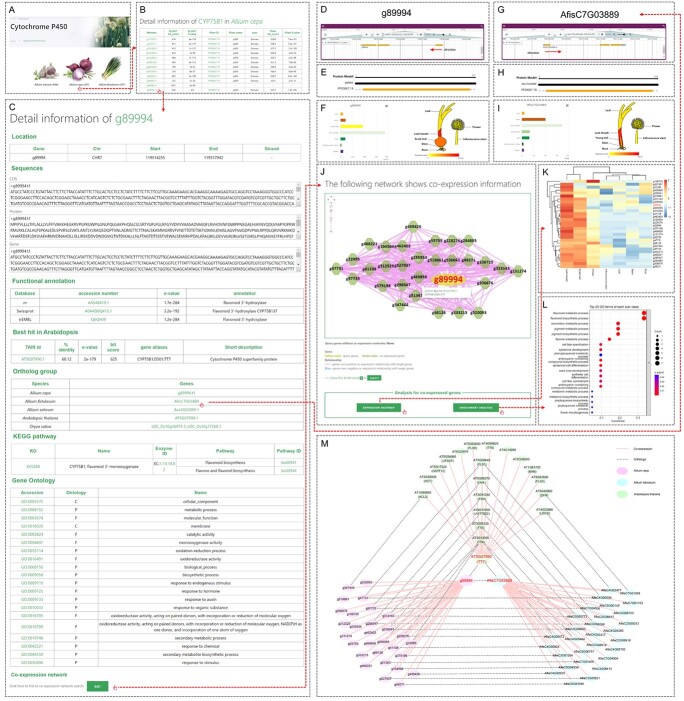
Functional and conservation analysis for a *CYP75B* gene (g89994). **A** Screenshot of the browse page for CYP450s. **B** List of CYP75B members in onion (*A. cepa*). **C** Screenshot of the gene information page for g89994. **D** Gene structure of g89994. **E** Relative location of Pfam domains in the protein encoded by g89994. **F** Expression pattern of g89994 in different tissues. **G** Gene structure of AfisC7G03889. **H** Relative location of Pfam domains in the protein encoded by AfisC7G03889. **I** Expression pattern of AfisC7G03889 in different tissues. **J** Screenshot of the search result for the co-expression network of g89994. The green polygons are genes co-expressed with the search gene (shown in yellow). The pink lines indicate positive co-expression relationships. **K** Expression pattern of genes co-expressed with g89994 in different tissues. **L** Top 20 enriched GO terms among the genes co-expressed with g89994. **M***CYP75B* network in *Arabidopsis*, *A. cepa*, and *A. fistulosum*. Functional partners of AtCYP75B1 [also named TRANSPARENT TESTA 7 (TT7)] in *Arabidopsis* were searched in the STRING database. The red font indicates *CYP75B*. Panels A–G are direct screenshots from AlliumDB.

The CYP450s are one of the largest families of enzymatic proteins in plants. CYP450s catalyze extremely diverse reactions involved in the biosynthesis of structural macromolecules, signaling molecules, pigments, and defense compounds [23]. We predicted all genes encoding CYP450s in *Allium*; these results are stored in AlliumDB (Fig. 3A). *Allium* bulbs are rich in flavonoids, which have antioxidant, anticancer, hypolipidemic, antidiabetic, cardioprotective, neuroprotective, and antimicrobial activities [24]. CYP75B [also named flavonoid 3′-hydroxylase (F3′H)], a CYP450 family member, catalyzes the 3′-hydroxylation of the flavonoid B-ring. In *A. cepa*, 12 CYP75B1 members were identified by BLAST search, 11 of which contain a P450 domain (PF00067.19) (Fig. 3B). In the gene information page for g89994 (Fig. 3C), the *CYP75B1* gene with highest bit score, its functional annotation is consistent with the best ortholog hit in the Nr, Swiss-Prot, trEMBL, and TAIR databases and GO and KEGG annotations. These results indicate that the functional annotation of this gene is highly reliable. The ortholog in *A. fistulosum* predicted by OrthoFinder is AfisC7G03889 (Fig. 3C). The genes g89994 and AfisC7G03889 have similar gene structures and are preferentially expressed in leaf sheaths, suggesting that these two genes may share a certain degree of conservation (Fig. 3D–I).

We searched the co-expression network of g89994 to analyze the possible biological function and regulatory network of this gene (Fig. 3J). We identified 29 genes that are co-expressed with g89994 and preferentially expressed in bulb scales (Fig. 3K) and the pseudostem (leaf sheath). These co-expressed genes were particularly commonly associated with the GO terms ‘flavonoid biosynthetic process’ and ‘flavonoid metabolic process’, which is consistent with the catalytic function of CYP75B in *Arabidopsis* (Fig. 3L). To test the reliability of the co-expression network, we searched the functional network of the orthologous gene *AtCYP75B1* [also named *TRANSPARENT TESTA 7* (*TT7*), At5g07990], whose encoded protein shows 60.5% identity with the protein encoded by g89994, in the STRING database [25] (Fig. 3M). We detected orthologs for 8 of the 29 genes co-expressed with g89994 in the STRING network for *AtCYP75B1*, including *FLAVANONE 3-HYDROXYLASE* (*F3H*), *TRANSPARENT TESTA 4* (*TT4*), *TT5*, *FLAVONOL SYNTHASE 1* (*FLS1*), *CHALCONE ISOMERASE LIKE* (*CHIL*), and *UDP-GLUCOSYL TRANSFERASE 78D2* (*UGT78D2*). These results underscore the high confidence for the co-expression network of g89994. In addition, we compared the co-expression networks of g89994 with that of its orthologous gene AfisC7G03889 in *A. fistulosum*, whose encoded protein shares 96.1% identity with that encoded by g89994. We identified orthologs for 13 of the 29 genes co-expressed with AfisC7G03889 in the network for *A. fistulosum*, indicating that the co-expression networks of *CYP75B* are conserved between *A. cepa* and *A. fistulosum* (Fig. 3M).

In addition, the g89994 co-expression network was enriched in GO terms related to the biosynthesis and metabolism of pigments, flavones, phenylpropanoids, and anthocyanin. CYP75Bs affect pigment biosynthesis in other species [26, 27]. Of the genes co-expressed with g89994, the transcription factor-encoding genes g49271 (WRKY), g104504 [basic helix–loop–helix (bHLH)], g312529 (MYB), and g106725 (WRKY) showed particularly strong positive co-expression with g89994. The WD-repeat–bHLH–MYB complex acts as an important regulator of anthocyanin accumulation [28–30]. AcB2, which is identical in sequence to the protein encoded by g104504, interacts with AcMYB1 (itself identical to g312529) to induce anthocyanin accumulation in the epithelial cells of onion bulbs [31, 32]. These findings suggest that these co-expressed transcription factor genes might regulate flavonoid biosynthesis to influence bulb color, which is an important consumer trait for onions. Furthermore, AfisC6G01045 (WRKY) and AfisC5G04934 (bHLH) are the orthologs of g49271 and g104504, respectively, and are co-expressed with *CYB75B* in *A. fistulosum*. This finding suggests that WRKY and bHLH may play similar regulatory roles in *A. fistulosum* compared with *A. cepa* (Fig. 3M). The co-expression networks available at AlliumDB, based on large-scale transcriptome data integration, thus provide new insights for functional and regulatory studies.

## Conclusions and future directions

In summary, AlliumDB contains the most comprehensive genomes, sequence annotations, genome comparisons, genome variations, transcriptomes, proteomes, and phenotypes of germplasms to date from multiple worldwide *Allium* species. These important and rich datasets can be useful for understanding gene function and molecular mechanisms through the exploration of genomes, variations, gene expression, and phenotypes, facilitating the development of future optimal breeding strategies. AlliumDB provides a concise and comfortable interface, user-friendly search and browse functions, practical analysis tools, a convenient download center, and various types of visualization displays. AlliumDB is designed with quick links to facilitate interactions between each function. We believe that AlliumDB will be extremely useful for researchers and breeders looking to fully utilize complex and rich omics datasets for fundamental functional investigations and molecular breeding of *Allium* crops.

With the decreasing cost and growing power of sequencing, the genomics resources available for *Allium* species are increasing rapidly. In the future, we will continue to update AlliumDB as new genomes and omics data become available. AlliumDB will be updated continuously with phenotypic data for *Allium* germplasms, which our group scores manually every year. Furthermore, we are assembling the genomes of onion and Welsh onion and generating large-scale whole-genome resequencing data for all *Allium* germplasms described in AlliumDB. We plan to add more variation data to the database, including SNPs, insertion/deletion (InDel) polymorphisms, and structural variations (SVs), to facilitate the determination of associations between genotypes and phenotypes in the future. We will also add more annotations and functionalities to the database, such as multi-omics data (e.g. epigenomes, proteomes, metabolomes, and phenomes) and tools for deep mining of multi-omics data, as well as molecular marker resources for genomic breeding. AlliumDB will thus provide long-term support to the *Allium* research community.

## Materials and methods

### Data sources

The *Allium* nuclear genome data, including whole-genome sequences, cDNAs, protein sequences, and annotation files, were obtained from previous studies [5–7] (Supplementary Data Table S1). The organelle genomes were downloaded from the National Center for Biotechnology Information (NCBI) GenBank database (Supplementary Data Table S2). Maps of organelle genomes were downloaded from OrganellarGenomeDRAW [33]. Diverse types of omics data, including RNA-seq and whole-genome resequencing data, were downloaded from the NCBI SRA database (www.ncbi.nlm.nih.gov/sra) and NGDC-CNGB (https://ngdc.cncb.ac.cn/) (Supplementary Data Tables S3 andS4). The genomes of the model species *Arabidopsis* (TAIR10) and rice (MSU7) were downloaded from TAIR (http://www.arabidopsis.org/) and the Rice Genome Annotation Project (RGAP) (http://rice.uga.edu/), respectively. Annotation information was collected from the Nr, Swiss-Prot, trEMBL, TAIR, Pfam (http://pfam.xfam.org/), and Rfam (https://rfam.org/) databases. Motifs were downloaded from the JASPAR database (https://jaspar.genereg.net/) for the motif scan.

### Functional annotation

Orthologous pairs of sequences between *Allium* species, *Arabidopsis* (TAIR10), and rice (MSU7) were identified using OrthoFinder software [34]. The best hits in the Nr, Swiss-Prot, trEMBL, and TAIR databases were identified using BLASTp [35]. Protein domains were predicted using localized PFamScan software [36]. Enzymes and metabolic pathways in the *A. fistulosum* genome were annotated using KofamKOALA [37] from the KEGG database. Genes from the *A. fistulosum* and *A. cepa* genome were annotated with GO terms using Blast2GO [38]. The KEGG and GO annotations in *A. sativum* were extracted from functional annotation files of the corresponding genomes [5]. The ncRNAs in the three *Allium* species were predicted using cmscan from the Infernal software [39].

### Gene family classification

Transcription factors or regulators and protein kinases were predicted using the iTAK tool (http://bioinfo.bti.cornell.edu/cgi-bin/itak/index.cgi) with data from PlnTFDB [40]. Cytochrome P450s were predicted from the best hits of protein BLAST searches using protein sequences from http://drnelson.uthsc.edu/CytochromeP450.html. Flavor-related genes were collated from the literature [7].

### Resequencing data processing

The raw reads from *A. fistulosum* were downloaded from NGDC-CNGB (accession CNP0002276). Low-quality resequencing reads were removed using fastp software (version 0.23.1) (https://github.com/OpenGene/fastp). The resulting high-quality reads were aligned to the *A. fistulosum* genome using BWA-MEM [41] with default parameters. The alignment results were then converted into BAM format and sorted using SAMtools [42], which was also used to remove duplicate PCR reads. Subsequently, the genomic variants for each sample were identified using BCFtools (https://github.com/samtools/bcftools). The variants were further filtered using the following criteria: depth for each individual <3 and mapping quality <20.

### Genome-wide association study

GWAS was re-analyzed using the Efficient Mixed Model Association Expedited (EMMAX) algorithm [43]. Population stratification and hidden relatedness were determined using a kinship (*K*) matrix, which was generated by the emmax-kin program. The *P*-value threshold for suggestive associated loci was set according to the estimate from a Bonferroni correction based on the effective number of independent markers [44].

### Germplasm data

A total of 423 *Allium* germplasms were collected from 33 countries: 250 (61%, from 12 countries) from Asia, 109 (27%, from 15 counties) from Europe; 48 (12%, from 4 countries) from the Americas; 1 each from Sudan and New Zealand; and 14 with unknown regions (Supplementary Data Table S5). Photographs of *Allium* germplasms were taken, and nine traits (tillering, leaf wax, bolting, leaf shape, leaf gesture, orientation of leaf, node density, length of pseudostem, germination rate) were scored.

### RNA-seq of *A. cepa* and *A. fistulosum* from different tissues

A total of 543.43 Gb RNA-seq data were acquired from 49 samples of *A. cepa* and *A. fistulosum.* Seven representative tissues of each of *A. cepa* and *A. fistulosum* were selected. For *A. cepa* the tissue samples comprised flower, inflorescence stem, leaf, leaf sheath, stem, root, and scale leaf, and each tissue was collected as three replicates. For *A. fistulosum* the tissue samples consisted of flower, inflorescence stem, leaf, leaf sheath, stem, root, and young leaf, each tissue being collected as four replicates (Supplementary Data Table S3). A library was prepared and sequencing was performed on an Illumina NextSeq instrument to generate 150-bp paired-end reads by the company Annoroad Gene Technology Beijing Co. Ltd.

### RNA-seq data processing

FastQC software (http://www.bioinformatics.babraham.ac.uk/projects/fastqc/) was used for quality control. RNA-seq reads from *A. cepa*, *A. sativum*, and *A. fistulosum* were aligned to the corresponding reference genome [5–7] using HISAT2 software with default settings [45]. The gene expression levels were normalized as FPKM values using StringTie software with default settings [46]. Tissue-specific genes were identified by FPKM values from in-house RNA-seq data using TBtools software [47].

### Co-expression network construction

The FPKM values for all samples in a species (Supplementary Data Table S3) were used to calculate Pearson’s correlation coefficient (PCC) and mutual rank (MR) values, which were used to construct global gene co-expression networks as previously described for *Arabidopsis* [48], cotton (*Gossypium hirsutum*) [49], and wheat (*Triticum aestivum*) [50]. Considering the coverage and connectivity of the networks, positive and negative gene pairs were retained when the corresponding |PCC| ≥ 0.6 and MR ≤ 30.

### *De novo* transcriptome assembly

Trinity software (https://github.com/trinityrnaseq/trinityrnaseq) was used for transcriptome assembly for a single species with default settings. To generate effective unigenes, Corset software (https://github.com/Oshlack/Corset), with default parameters, was used to exclude redundant data. Transcriptome completeness was assessed by calculating the Benchmarking Universal Single-Copy Orthologs (BUSCO, https://busco.ezlab.org/) score to obtain the percentage of single-copy orthologs using the dataset embryophyta_odb10. Functional annotations of unigenes were conducted using eggNOG-mapper (http://eggnog-mapper.embl.de/).

### Collinear block identification

MCScanX [51] was used to extract collinear blocks from the alignment results. Alignments of amino acid sequences of all proteins encoded by each gene were generated using BLASTp [35] with an E-value threshold of 1e−5. The general feature format (GFF) files and BLAST output files of all protein-coding genes were imported into MCScanX to scan for collinear pairs (a minimum of five genes were required to call a collinear block).

### Toolkit development

All genomic features and omics data were visualized using JBrowse [52] and its plugins. The networks were displayed using cytoscape.js (http://js.cytoscape.org/). Synteny visualization was provided by SynVisio (https://synvisio.github.io/#/) and circosJS (https://github.com/nicgirault/circosJS). The Manhattan plots for GWAS were generated using ECharts javascript (https://echarts.apache.org/). The bar charts of FPKM values were generated using Highcharts javascript (https://www.highcharts.com/). The expression heat map analysis was provided using the R package pheatmap (https://github.com/raivokolde/pheatmap). The GO, KEGG, and Geneset enrichment analysis was performed using R package clusterProfiler [53]. Sequence extraction based on location was provided by BEDtools [54]. Motif analysis was performed using MEME Suite (https://meme-suite.org/meme/).

### Database construction

The database was constructed based on a standard LAMP (Linux + Apache + MySQL + PHP) system. The datasets are stored in MySQL (www.mysql.com) and formatted text files. The interactive web pages were implemented using the HTML, CSS, JavaScript, and PHP languages (www.php.net) in Red Hat Linux powered by an Apache server (www.apache.org). The database is freely accessible to users for academic purposes, and there are no login requirements.

## Supplementary Material

Web_Material_uhad285
